# Ni/Al-Hybrid Cellular Foams: An Interface Study by Combination of 3D-Phase Morphology Imaging, Microbeam Fracture Mechanics and In Situ Synchrotron Stress Analysis

**DOI:** 10.3390/ma14133473

**Published:** 2021-06-22

**Authors:** Jutta Luksch, Anne Jung, Christoph Pauly, Ralf Derr, Patrick Gruenewald, Marc Laub, Manuela Klaus, Christoph Genzel, Christian Motz, Frank Mücklich, Florian Schaefer

**Affiliations:** 1 Materials Science and Methods, Campus D2 3, Saarland University, 66123 Saarbruecken, Germany; j.luksch@matsci.uni-sb.de (J.L.); p.gruenewald@matsci.uni-sb.de (P.G.); m.laub@matsci.uni-saarland.de (M.L.); motz@matsci.uni-sb.de (C.M.); f.schaefer@matsci.uni-sb.de (F.S.); 2 Applied Mechanics, Campus A4 2, Saarland University, 66123 Saarbruecken, Germany; r.derr@mx.uni-saarland.de; 3 Functional Materials, Campus D3 3, Saarland University, 66123 Saarbruecken, Germany; c.pauly@mx.uni-saarland.de (C.P.); muecke@matsci.uni-sb.de (F.M.); 4 Department of Microstructure and Residual Stress Analysis, Helmholtz-Zentrum Berlin fuer Materialien und Energie GmbH, Albert-Einstein-Str. 15, 12489 Berlin, Germany; klaus@helmholtz-berlin.de (M.K.); genzel@helmholtz-berlin.de (C.G.)

**Keywords:** hybride foam, open-cell foam, interfacial fracture toughness, micromechanics, synchrotron diffraction, hybride foam interface

## Abstract

Nickel(Ni)/aluminium(Al) hybrid foams are Al base foams coated with Ni by electrodeposition. Hybrid foams offer an enhanced energy absorption capacity. To ensure a good adhering Ni coating, necessary for a shear resistant interface, the influence of a chemical pre-treatment of the base foam was investigated by a combination of an interface morphology analysis by focused ion beam (FIB) tomography and in situ mechanical testing. The critical energy for interfacial decohesion from these microbending fracture tests in the scanning electron microscope (SEM) were contrasted to and the results validated by depth-resolved measurements of the evolving stresses in the Ni coating during three-point bending tests at the energy-dispersive diffraction (EDDI) beamline at the synchrotron BESSY II. Such a multi-method assessment of the interface decohesion resistance with respect to the interface morphology provides a reliable investigation strategy for further improvement of the interface morphology.

## 1. Introduction

Foams are bio-inspired, cellular and lightweight materials that offer a unique energy absorption capacity e.g., as crash energy absorber [1,2,3,4] or also as catalyst supports [5]. Hybrid foams belong to the group of composite foams and are characterized by a combination of different materials. Typical hybrid foams are copper (Cu)/Al hybrid foams [6,7,8] and Ni/polyurethane (PU) hybrid foams [9]. Ni/Al hybrid foams are made from an Al base foam. The nanocrystalline Ni coating is applied electrochemically [10,11,12,13]. Such foams offer better mechanical properties compared to standard open-cell foams of comparable density.

The aim of this study is the direct comparison of two states of an Al base foam with regard to the effect on the mechanical performance of the hybrid foam. Either the Al base foam was subjected to a chemical pre-treatment or the Ni coating was electroplated directly to the Al base foam.

Considering the mechanical properties, cellular solids can be divided into three different hierarchical scales [14,15]. While the macroscale involves complete foam components, the mesoscale is determined by pores, while the microscale contains individual struts characterized by their microstructure [16]. That macro-meso-micro-(MMM)-principle is helpful in further improving microheterogeneous materials.

Resulting from this MMM-principle, the micromechanical properties of the form determine the global material behaviour. In particular, the energy absorption efficiency is less influenced by the strut geometry rather than the mechanical properties of the strut [17,18,19]. Pronounced variations in the mechanical behaviour of foams on the macroscale can be attributed to differences on the mesoscale as well as on the microscale. The latter are effected by deviations in the dimensions and mechanical properties of the individual struts. Bending and buckling of the struts is considered the main failure mechanism of foams [20]. The struts have fixed boundary conditions via the pore network.

**Figure 1:**
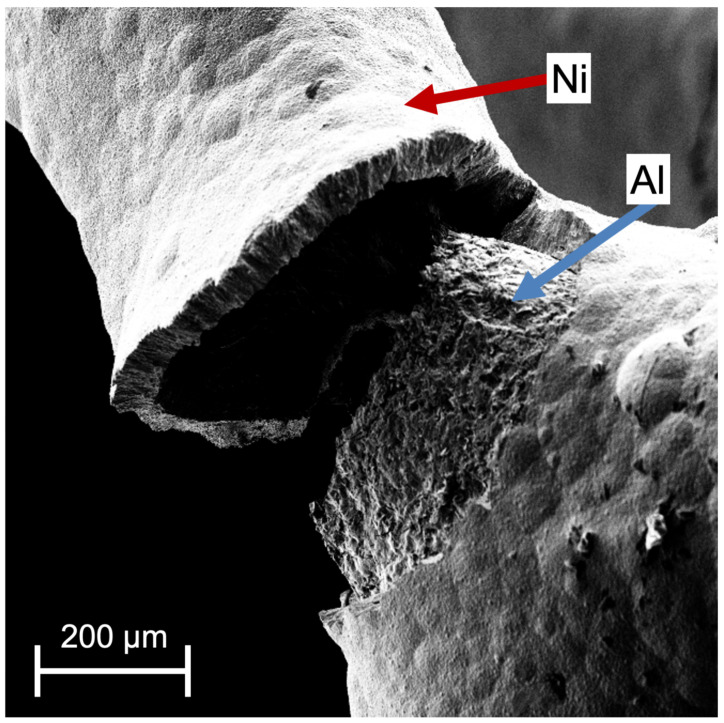
Ni/Al hybrid foam strut after in situ compression of a single pore in SEM, delamination of the Ni coating is clearly visible.

Especially in hybrid foams the interface between two materials is exposed to very large stress due to high tangential forces during bending. The buckling resistance of its struts is directly linked to the strength of the connection between the coating layer and the base material. In previous work in situ SEM experiments at single pores of Ni/Al hybrid foams under compression revealed failure at the Ni/Al interface (Figure 1). Sun et al. found a degradation of the compression performance for over-annealed Al/Cu hybrid foams coincident with the formation of intermetallic precipitates at the interface between Al and Cu [21]. Cho et al. [22] revealed by finite element method (FEM) simulations that a debonded hybrid foam interface results in a reduced elastic modulus. A lower elastic modulus promotes bending and buckling of struts resulting in a reduced compressive strength. Liu et al. concluded that interface debonding is a main failure mechanism during compression of hybrid foams at room temperature and at 200 °C [9].

In order to address the weak point interface, it is important to understand the failure mechanism in view of the interface morphology. Interface debonding has been identified as a main failure mechanism of struts that leads to larger bending and premature buckling. However, the complex interplay between the interface morphology and the strength of the interface cohesion needs further and detailed investigation.

Micromechnical experiments are an expedient method to precisely probe the mechanical behaviour of the Ni/Al interface. By in situ SEM tests of small structures size effects during plastic deformation [23,24,25,26], strain induced grain growth [27] and the fracture toughness of small specimens [28,29,30,31] have been evaluated. The target preparation enables to even analyze the fracture resistance of single grain boundaries [32,33].

In order to assess the interface properties of a Ni/Al hybrid foam the interface was analyzed with three different methods:

- First, a tomography of the interface was conducted of the two different states of the Ni/Al hybrid foam for comparison: as already mentioned above, one Al base foam had been chemically pre-treated prior to electroplating and one was coated without a pre-treatment. A tomography by FIB proved to be a powerful tool to reveal complex 3D microstructures and interface morphologies, even on the nanoscale, that are inaccessible in 2D [34,35,36].
- In a second step the evolution of the in-plane stress distribution perpendicular to the bending axis was measured in the Ni coating of individual struts by depth-sensitive X-ray diffraction (XRD) at EDDI beamline at the synchrotron storage ring BESSY II in Berlin. The special setup at the EDDI beamline allowed to achieve a complete diffraction stress analysis by sin^2^*ψ*-method with high depth-resolution applied inter alia for thin coating layers [37]. A specially adapted in situ three-point bending testing rig was used. The critical load needed for decohesion of the Ni coating, measured directly at the interface, was compared for the two pre-treatment states of the foam.
- In a third and concluding observation micro bending beams including the Ni/Al interface were cut with FIB. The interface was positioned to the maximum stressed volume during cantilever bending. The critical energy for interfacial crack growth was identified for the two states of pre-treatment.

## 2. Materials and Methods

### 2.1. Interface Manufacturing

Al alloy foams (AlSi_7_Mg·3) produced by investment casting based on a template analogous to [38] with a pore size of about 10 ppi manufacturer information were purchased from Celltec Materials GmbH (Dresden, Germany) and coated with a hard facing Ni layer using electrodeposition [12,13] with a coating thickness of about 50 μm. For the electrodeposition a commercial Ni sulfamate electrolyte at a pH of 3.8 and a temperature of 40 °C was used as described by Jung et al. [12].

**Figure 2:**
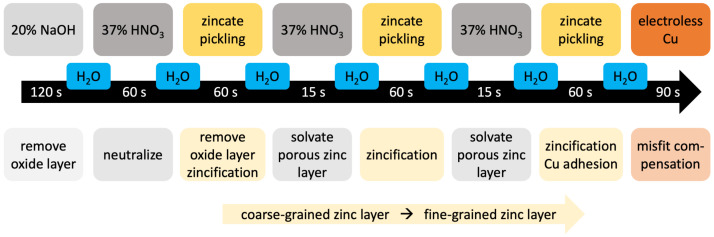
Schematic of the pre-treatment process and the aim of each step.

Prior to the Ni deposition a chemical pre-treatment is applied onto the Al foam as outlined by Jung et al. [39]. The pre-treatment consists of eight steps (Figure 2): After an alcalic cleaning with NaOH an acidic cleaning with HNO_3_ follows with a subsequent zinc (Zn) pickling (75 g/L ZnO and 350 g/L NaOH) with NaOH for neutralization. The HNO_3_ cleaning and the Zn pickling are repeated twice. The resulting Zn layer ensures that the Al foam does not dissolve in the acid electrolyte. Finally a Cu layer (150 g/L CuSO_4_-5H_2_O and 50 g/L H_2_SO_4_) is applied by electroless plating onto the Zn layer. The Cu layer has as the Zn layer a a vanishing thickness and adjusts the mismatch in the atom radius between Al and Ni and the coefficient of thermal expansion for better adhesion of the Ni deposition.

The pre-treatment is expected to improve the adhesion of the subsequent electrochemical Ni coating to the Al foam as described in details in Jung et al. [12] by an improved interlocking between coating and base foam. To investigate whether this time-consuming procedure provides benefits for the mechanical behaviour of the foam and to study the mechanisms behind the adhesion of the Ni coating as far as to find approaches for further improvement of the pre-treatment, struts from pre-treated and untreated Al foams are analyzed after coating with Ni.

For the following experiments single struts were cut out in a two step process. Diagonal pliers were used to avoid damage of the microstructure. First, a piece of about one to two cubic centimetre, comprising several struts, was cut out. Finally, the recent struts were cut of, leaving a whole single strut. That strut with the two nodes had an approximate size of 2–4 mm length and 0.6–0.8 mm width.

All tests described below were performed at room temperature.

### 2.2. Interface Morphology

For a target preparation, first the extracted struts from a pre-treated and from an untreated sample were embedded into the dissolvable investment resin Technovit 5075, Kulzer GmbH (Hanau, Germany), and then ground and polished perpendicular to the interface along the length axis of the beam. The resin was removed with acetone.

A representative area on the interface was chosen for the subsequent FIB tomography. As preparatory steps, platinum (Pt) was deposited on a representative 10 × 10 μm^2^ area to prevent gallium (Ga) implantation and curtaining.

Secondary electron images (acceleration voltage 5 keV) were acquired with a Helios NanoLab 600 SEM (FEI, Hillsboro, OR, USA) with a slice thickness of 30 nm. The 3D volume reconstruction from the tomography was conducted with the software tool Amira (also from FEI). Due to shading and edge effects at the pores the segmentation was done manually to ensure accurate results for the 3D volume reconstruction.

The evaluation of the volume fraction of phases and of the area of the interfaces was done with the software MAVI from Fraunhofer Institute for Industrial Mathematics ITWM (Kaiserslautern, Germany).

### 2.3. Stress Distribution Analysis with Energy-Dispersive X-ray Diffraction

**Figure 3:**
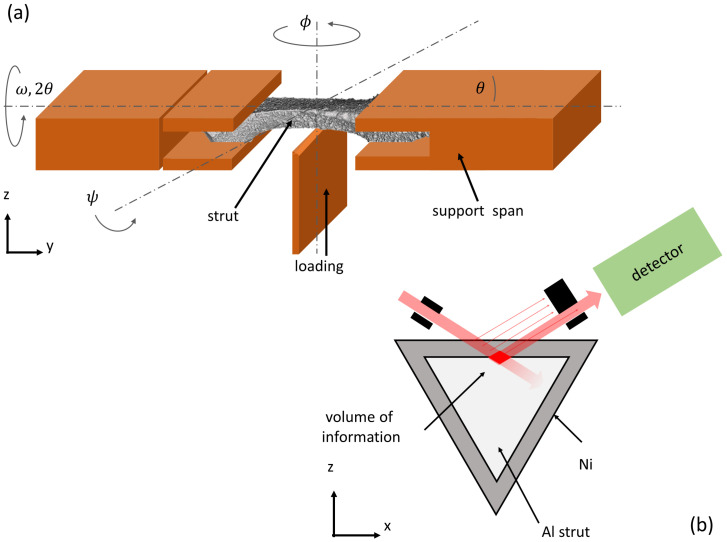
Schematic representation of the experimental setup. (**a**) The strut is fixed by the support span. The loading direction is positive vertical and the analysis is performed below the upper surface the interface using an immersed beam and narrow slits. (**b**) Schematic cross section through the middle of the strut. The slit and detector setup and the beam at fully immersed position is shown.

**Figure 4:**
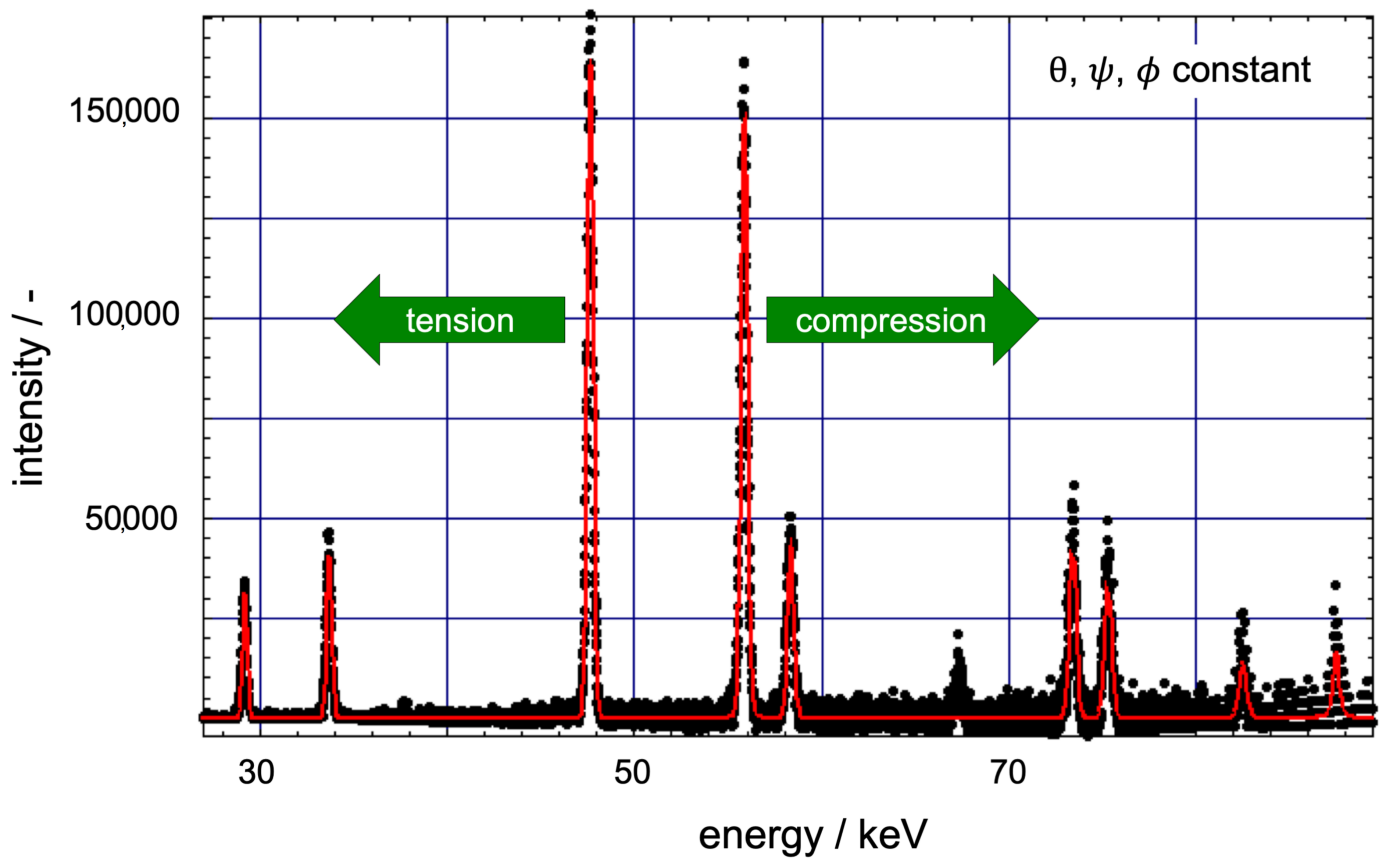
Typical energy spectrum with diffraction peaks: the peaks are shifted to the right in case of compression of the diffracting lattice planes and to the left in case of expansion or tension. Each peak has a specific information depth due to its energy.

The energy-dispersive (ED) method provided at the EDDI beamline allowed for a depth-resolved analysis. The residual stress values for the in-plane bending stress $\sigma_{xx}$ regarding the coordinate system in Figure 3 and the shear stress $\sigma_{xy}$ obtained by applying the sin^2^*ψ*-method to the individual reflections *hkl* in the ED diffraction patterns could be assigned to different average information depths. The stresses were calculated according to previous work of Genzel et al. [37,40,41,42]. Compression of the analyzed volume led to a shifting of the peaks to a higher energy and tension to a lower energy (Figure 4). The lateral resolution, on the other hand, was limited by the aperture of the slits that form the primary and the diffracted beam, respectively (Genzel et al. [41]).

An ED X-ray detector provides, in contrast to a time-consuming angular-dispersive (AD) strategy, a complete X-ray spectrum for a polycrystalline material, whereas each peak corresponds to a distinct energy and therefore a characteristic information depth τ, with an intensity of 1/e defined as the information depth. This can be used to perform a time-efficient $d_{hkl}$-sin^2^*ψ*-analysis of each peak with a high information depth due to the high brilliance and the higher energy (used practically up to 100 keV) of synchrotron radiation compared to conventional lab sources.

Special attention must be paid to the concurrence of geometric information depth arising from the cut gauge volume due to the slit setup (beam and detector side) and the 1/e information depth. A depth-resolved measurement uses the dependency of the specific mean information depth on the photon energy for each diffraction peak in the ED measurement. This results in a low lateral resolution because a large gauge volume must be ensured whereas a localized measurement can be achieved by small and narrow slit widths on both sides and an immersion of this very small gauge volume to the region of interest as the volume of information [40] (Figure 3).

For the preparation, the extracted struts were also embedded in a resin to avoid pre-deformation during grinding to the final size. After finishing the preparation the resin was removed with acetone. The struts offer a triangular cross section with a nearly flat side face of at least 1500 μm × 500 μm.

The in situ three-point bending tests were performed under displacement control with a Kammrath and Weiss 200 N testing device (Dortmund, Germany) using a specially designed fixture for the single strut specimens to avoid shadowing of the synchrotron white beam (Figure 3). The fixtures had a support span of 2 mm and rounded edges to reduce frictional effects. Special attention was paid to the alignment of the struts. The struts had two supports on the nearly flat side, where stresses are measured and the load applying rounded wedge on the apex.

Load increase tests were conducted and the evolving stress state in the Ni coating was monitored with a focus on the in-plane stress perpendicular to the bending axis because the strongest effects were expected for this component of the developing stress tensor from the FE-simulations shown later.

To achieve a depth-resolved measurement of the stress distribution in the Ni coating, a beam slit size of 100 μm × 100 μm was applied with a conventional slit system to reach a height of the geometric information depth larger than the coating thickness of 50 μm (Laplace method according to [42]). The detector slit size was adjusted to 30 μm × 8 mm. These depth-resolved measurements were conducted at a 2θ angle of 12° and an ω angle of 6°.

A 20 μm × 30 μm beam was provided by an elaborated narrow slit design [40] for the stress measurement at the interface. Hence, the in-plane spot size on the side face of a strut was about 300 μm × 30 μm at an ω-2θ configuration with a 2θ angle of 8°. The specimen was fully immersed in the beam to the Ni/Al interface at a 50 μm offset from the strut surface with a total height of the information volume (gauge volume) of less than 40 μm with less than 20 μm in the Ni coating due to the data collection geometry.

The energy range of the synchrotron radiation was not restricted for all measurements.

At a defined 2θ angle, no Al peaks were found in the measured energy spectra because the Al foam is coarse-grained with a mean grain size larger than 1 mm in contrast to the nanocrystalline Ni coating.

The six struts investigated were analyzed after failure by SEM imaging. To get a better view of the interface near the failure zone, the struts were embedded in a resin again, slightly ground and polished only to remove material in the order of few microns. The cross sections were investigated by optical microscopy.

### 2.4. Microbeam Bending

**Figure 5:**
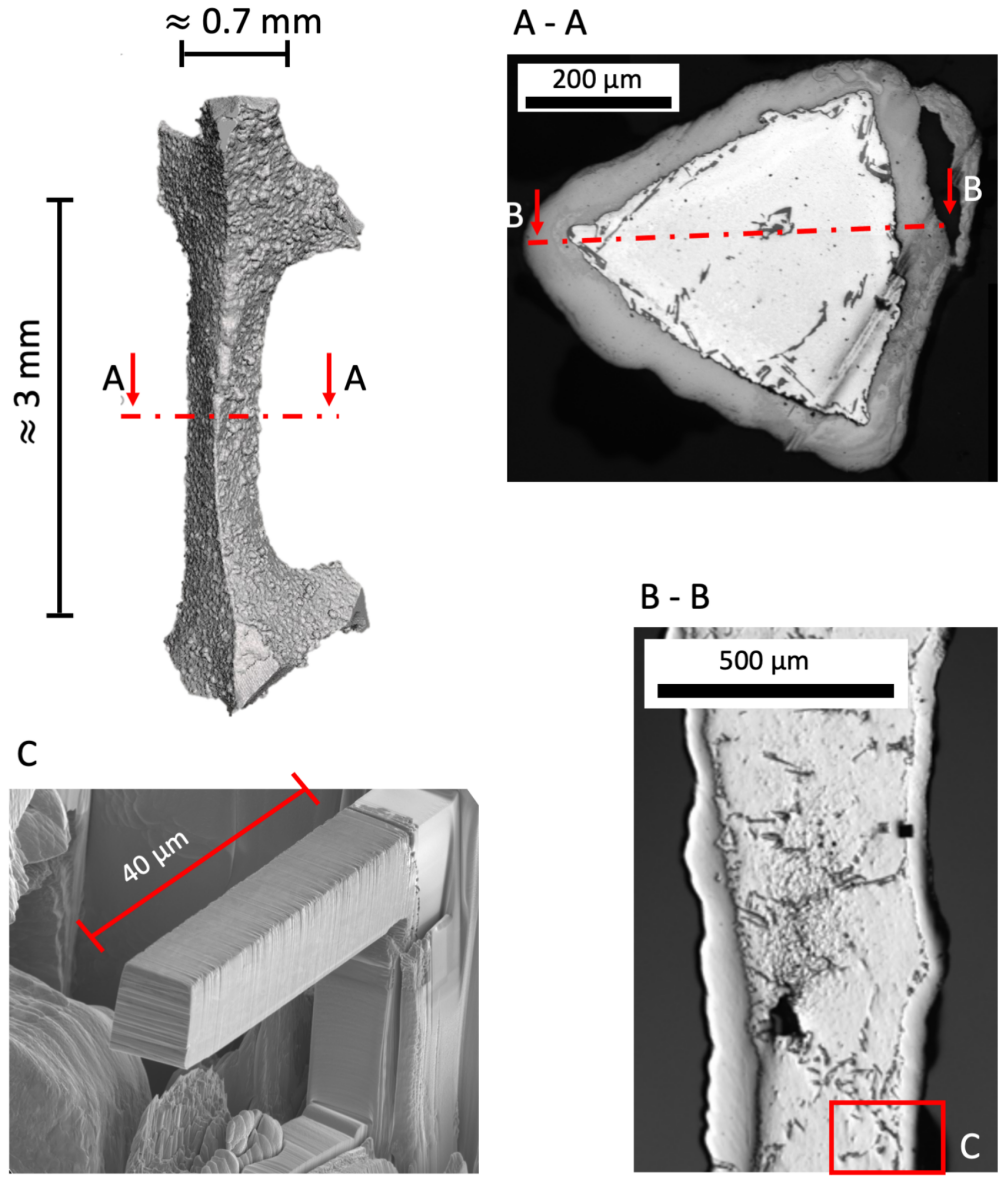
Positioning of microbending beam at the interface. The capital letters **A** and **B** indicate the location of the corresponding cross sections in the following sectional views **A-A** and **B-B**. Figure **C** shows detail **C** in cross section **B-B**. The location and orientation of the beam is depicted by cross-sectional images.

As for the sample preparation for the tomography already explained above, struts from a pre-treated and an untreated foam were also ground and polished for further FIB preparation. The interface is just at the shoulder position of the FIB cut beam (Figure 5). The beams were cut with a radius at the fixture of these cantilevers, because linear elastic FEM simulations revealed beforehand that a clearly localized stress maximum evolves at this position during bending. First, the beams were cut using a current of 21 nA and cut to the final size by a stepwise smaller ion current up to the final current of 0.48 nA. That stepwise procedure is necessary to keep Ga implantation low and obtain a good surface quality of the bending beam.

A notch was pre-cut perpendicular to the load axis from the side face of the beam with an ion current of 0.48 nA in a first step. In a second step, a sharpening of the notch with an ion current of 48 pA was performed from the upper side of the beam. The notch depth is 1.5 μm and due to the two step process the notch radius is expected to be less then 100 nm. This procedure ensures that the maximum stress is concentrated at the interface.

The bending tests were performed using a in situ nanoindenter device UNAT 2 (Asmec/Zwick Roell, Dresden, Germany). For good positioning of the tip on the beam and observation during the bending the tests were performed in situ in an SEM SigmaVP from Carl Zeiss AG (Oberkochen, Germany). The indenter tip was a FIB cut tungsten carbide wedge which applied the force with a lever arm of 30 μm.

A displacement controlled load function with partial unloading segments was applied to monitor crack growth referring to the J-integral evaluation according to ASTM E1820 [31]. Every 400 nm an unloading of 200 nm was conducted. After a force maximum followed by a significant force drop the experiment was finished.

It was deliberately decided not to aim for a J-integral evaluation. On the one hand, the notch position cannot exactly match the non-planar interface. On the other hand, the specimens were only notched, not cracked. This might lead to an overestimation of fracture toughness in the measurement results [31]. Furthermore, at an interface, the elastic part of *J* becomes infinite or vanishes due to inhomogeneity of the bending stiffness and the evolving incompatibility stresses [43]. Therefore, the evaluation was restricted to the measurement of the energy and just the analogy to the J-integral was pointed out.

For the data evaluation the slightly different cross-sectional areas of the beams due to the FIB preparation was taken to normalize the measured force to make the just slightly different beams completley comparable. With a co-simulation in ABAQUS^®^ from Dassault Systémes (Vèlizy-Villacoublay, France) the dependency of the beam bending compliance from the crack length was determined. The crack length was then calculated from the experimentally measured compliance, derived from several partial unloading segments. The energy necessary for further crack growth was calculated via the area underneath the normalized force vs. displacement curve for every unloading step. A more detailed description of the preparation and this evaluation can be found at Luksch et al. [44] and Gruenewald et al. [31]. Finally, energy vs. crack length diagrams were determined.

## 3. Results

### 3.1. FIB Tomography

**Figure 6:**
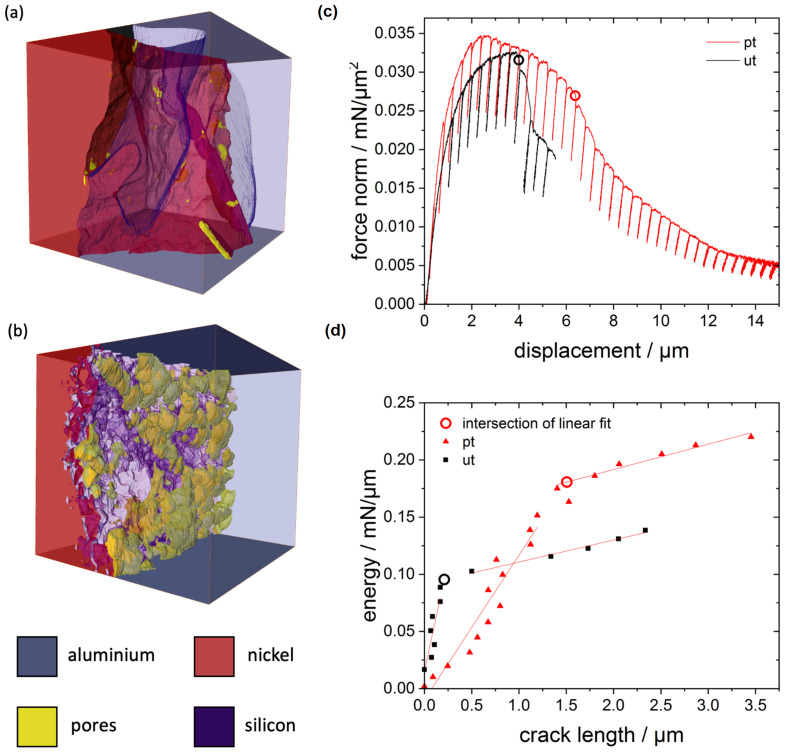
Results of the FIB-based evaluation methods: A visual comparison of the interface morphology of (**a**) an untreated sample and (**b**) a pre-treated sample from FIB tomography 3D reconstruction. Each volume is 8 × 8 × 8 μm^3^. On the right-hand side the results of the microbending experiments from a pre-treated (pt) and untreated (ut) specimen. (**c**) displacement vs normalized force with partial unloading segments. (**d**) Absorbed energy contrasted to the evolving crack length. (images adapted from [44]).

Representative volumes of 8 × 8 × 8 μm^3^ of the pre-treated and untreated strut interface are shown in Figure 6 after 3D reconstruction. In the volume of the untreated specimen a silicon precipitate was at the interface. However, the Al phase reached the Ni phase and an assessment of the interface morphology was possible. Obviously there were less and smaller pores in the untreated case and the interface was smoother than for the pre-treated case. The interface from the pre-treated volume was fragmented with large pore clusters resulting in a form-fit contact. The interface and the connection between Ni and Al seemed to be snap fastener-like. For better comparison between both cases, the shape parameters were evaluated as listed in Table 1.

The surfaces of the three phases, Al alloy, Ni and pores of the pre-treated specimen were more than three times larger. Especially the pore surface was more than 20 times larger than for the untreated specimen volume analyzed. The volume fraction of the pores in the evaluated volume was nearly 100 times higher for the pre-treated than for the untreated case. In order to elucidate the formation of the pores the shared surface of the pores with the Al and Ni phase was important. The shared surface of the pores with Al was 30 times larger than the one with Ni. This fact indicates that the pores were mainly on the interface at the Al side and resulted directly from the pre-treatment.

### 3.2. EDDI

For exemplary load steps, the in-plane shear stress $\sigma_{xy}$ was monitored but was about one magnitude lower compared to the in-plane stress $\sigma_{xx}$ perpendicular to the bending axis, although it was expected that the shear stress caused the delamination of the coating. The other components of the stress tensor were negligible.

**Figure 7:**
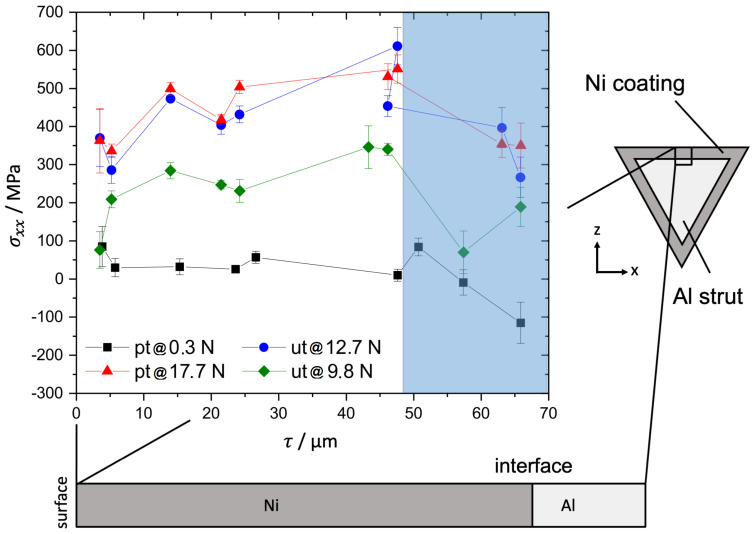
Depth distribution of the in-plane stress $\sigma_{xx}$ perpendicular to the bending axis measured by the depth-resolved technique: The untreated (ut) and the pre-treated specimen (pt) show a reduced in-plane stress perpendicular to the bending axis near the interface. Therefore, in case of zero applied stress, small compression residual stresses are present near the interface. The depth region of reduced local stress is marked in blue.

The depth-resolved stress measurements showed a small decrease in the in-plane stress perpendicular to the bending axis $\sigma_{xx}$ near the Ni/Al interface during all load steps for the pre-treated and the untreated hybrid foams (Figure 7). Hence, these measurements revealed a minor compression stress state after the electroplating that was not released under further deformation. This superimposed local compression stress state or the locally reduced stress near the interface should have had a positive effect on the interface stability during the deformation of the struts.

**Figure 8:**
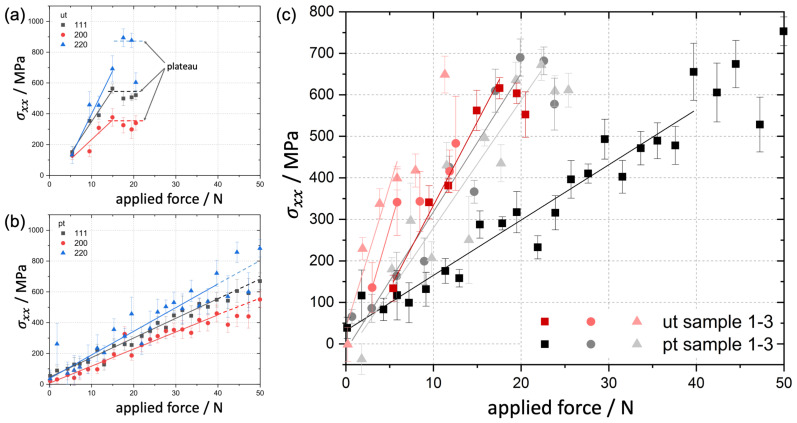
Measured in-plane stress perpendicular to the bending axis $\sigma_{xx}$ at the interface in Ni for different lattice planes for (**a**) an untreated (ut) and (**b**) a pre-treated (pt) specimen: the untreated specimens show a higher scattering of the data and a lower strength compared to the pre-treated ones. The data for the 6 investigated struts were gathered by full immersion of the beam to the Ni/Al interface (**c**).

The stress measurement with fully immersed beam to the interface showed that:

- Shear stresses were negligible. Only shear stresses of less than 20 MPa were measured at all load steps. This indicated a good homogeneity of the stress fields.
- If the applied load was increased the in-plane stress perpendicular to the bending axis also increased. At a level of 400 MPa up to 580 MPa the further capacity of the coating to accommodate stress was suddenly reduced and resulted in a plateau for the untreated foams. This stress region exhibited a large scatter for the untreated foams (Figure 8a).
- The pre-treated foam struts also showed a slight decrease of the further capable stress near the interface, without a real plateau, the critical values scattered less and the average value of this critical stress was about 100 MPa higher for the pre-treated struts (Figure 8b).

Figure 8 shows clearly the difference in the directional stress depending on the crystallographic direction. The stress was higher in the mechanical strong directions <220> and <111> compared to the soft directions <200>. The maximum load capable for the investigated six struts, three of each type (Table 2), differed due to the different cross-sectional area of each individual strut typical for the open cell foam. Therefore, no insight could be derived from the external applied failure load.

**Figure 9:**
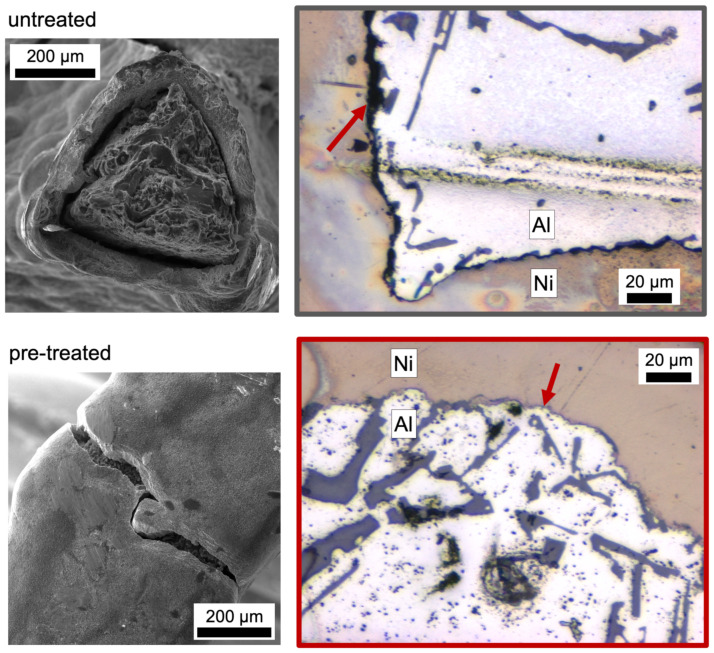
SEM secondary electron images of the struts after failure and cross sections near the fracture zone analyzed by optical microscopy after polishing: the decohesion in case of the untreated specimen (**grey**) is clearly visible whereas the pre-treated specimen (**red**) shows a fracture of the Ni coating but no visible decohesion in the cross section.

The post-mortem analysis of the failure region of all six investigated struts revealed a strong decohesion of the interface for the untreated foam and no visible decohesion near the fracture zone of the Ni coating for the pre-treated foam (Figure 9). Thus, it was deduced that the three struts without a pre-treatment were broken in the process zone whereas those with a pre-treatment showed only a fractured Ni coating.

### 3.3. Microbending Test

Figure 6 shows the normalized force vs. displacement curve of a pre-treated and untreated bending beam. The maximum normalized force was nearly the same for both beams, but the shape of the curves was different. The untreated beam showed a sudden load drop, while the pre-treated beam providd a slower decrease of the force after its maximum was reached.

From the normalized force vs. displacement curves energy vs. crack length curves were extracted. The curves, shown in Figure 6b, differed by the critical energy and the critical crack length, listed in Table 3. The untreated beam changed the slope in energy vs. crack length dependence already at half of the energy and seventh of the crack length compared to the pre-treated beam. The stable crack growth started earlier at $95.4\;\frac{J}{m^{2}}$ for the untreated case than for the pre-treated case at $180.7\;\;\frac{J}{m^{2}}$ (Table 3). This value, when transferred to a classical fracture toughness $K_{1C}$, could be estimated and assessed by the simple plane-stress equation $K=\sqrt{J\cdot E}$ to be less than 7 MPa$\sqrt{m}$ assuming that a mixed-Young’s modulus *E* of the bimaterial was between 50 and 200 GPa. Hence, the cohesion strength of the interface was very low compared to the typical bulk fracture toughness of Al and Ni.

**Figure 10:**
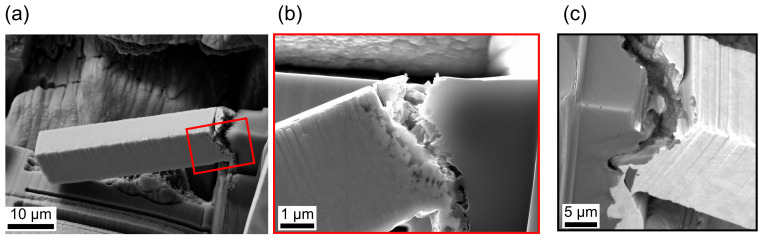
Post mortem SEM images from (**a**) pre-treated microcantilever with (**b**) a detailed view and (**c**) untreated microcantilever. The different crack path is visible.

From the in situ observation during the bending test via SEM and the post mortem images (Figure 10) a clear difference in the behaviour became obvious. The untreated beam failed along the interface. Hence, the fracture appeared mainly brittle. Considering the pre-treated beam, the fracture seemed to be ductile due to the large deformation besides the crack. Ultimately, failure occurred along the pores. Regarding the FIB cut notch, the untreated beam did not tear exclusively along the notch, but followed the interface different from the notch. Additionally, the crack of the pre-treated beam did not entirely follow the notch, but followed the weakest point of the interface, which seemed to be the pore network on the interface at the Al side. Due to the wavy interface it was not possible to pre-notch the beams right at the very curved interface.

## 4. Discussion

### 4.1. Morphology and Expected Material Behaviour

There are various mechanisms for bonding a coating to a substrate. In addition to a form-fit connection, also a material-fit and a force-fit connection are possible. For a coating, only the form-fit and material-fit connection types are worth considering. With FIB tomography, the connection was examined for possible form-fit effects.

Considering only the images from the FIB tomography, a clear difference in the two coating pre-treatment states is already obvious. The volume of the interface region of the untreated specimen shows a smooth surface with fewer and smaller pores, whereas the volume of the pre-treated specimen outlines a fragmented surface with bigger pore clusters in the range of a few μm. Due to the smooth surface, obviously there can only be a material-fit connection for the untreated specimen. In contrast, the morphology of the pre-treated specimen shows undercuts of Ni which leads to a form-fit connection and a more than three times larger shared surface between Al and Ni compared to the untreated specimen (Table 1). That larger shared surface of the pre-treated specimen enables also a larger area of material-fit connection between the coating and the foam strut.

However, the large pore clusters found in the pre-treated sample must also be considered. These might lead to a strong weakening of the connection. To what extent these aspects contribute to the stability of the connection, especially in the pre-treated case, is not clear from FIB tomography. Further evaluation steps are necessary for an assessment of the adhesion and the strength of the connection between coating and base foam.

### 4.2. Decohesion Stress Analysis

To better understand the measurement from the in situ XRD bending experiments, especially the formation of the plateau and the slight decrease in the capable stress at the interface previously, 2D-FEM simulations were performed using the software ABAQUS^®^ from Dassault Systémes (Vèlizy-Villacoublay, France). A straight strut cross section of 400 μm side face widths and a length of 1.2 mm was applied to three-point bending by an analytically rigid spherical indenter. A 50 μm Ni coating was tied to the Al base strut by a cohesive zone. The decohesion strength of the cohesive zone was modeled exemplary with 200 MPa under tension and 100 MPa under shear stress. To reveal the correct decohesion strength from the XRD measurements is not possible because the idealized strut geometry cannot reflect the complex 3D shape of the investigated struts. After the interface failure, a surface-to-surface hard normal contact and an exemplary tangential friction coefficient of 0.1 were implemented. The properties of the cohesive zone are model data and only serve to elucidate the effect of interfacial decohesion on the overall mechanical behaviour.

**Figure 11:**
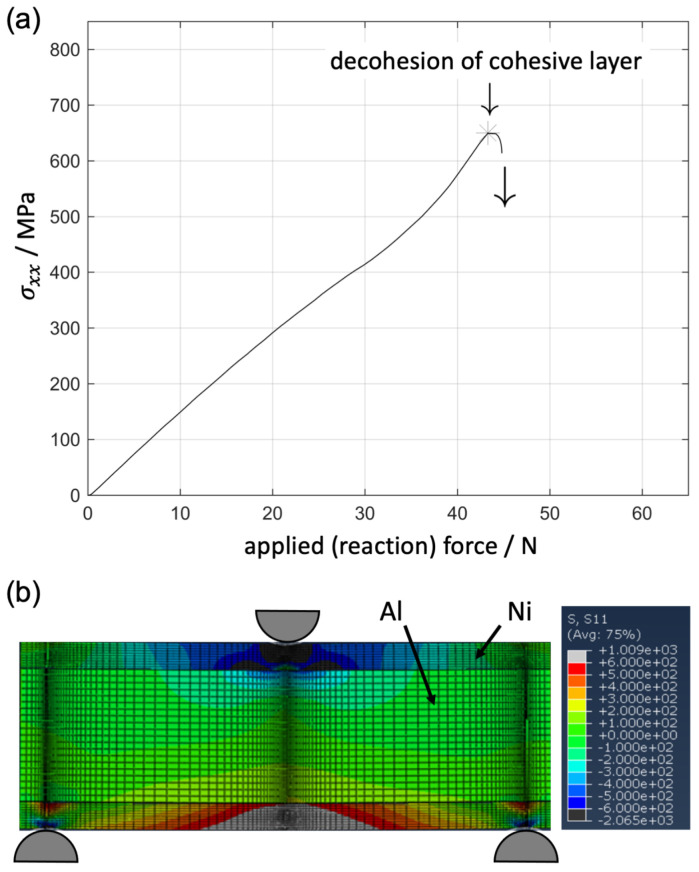
(**a**) Measured in-plane stress perpendicular to the bending axis at the Ni/Al interface vs. applied force (reaction force) from 2D FEM simulation, the moment of the decohesion of the interface modelled by a cohesive zone interlayer is marked (**b**) distribution of the in-plane stress perpendicular to the bending axis (limits +/− 600 MPa).

The material was modelled with isotropic elastic-ideal-plastic deformation. The simulations were performed with CPE4R elements in a displacement controlled step. Figure 11a shows the reaction force on the indenter versus the in-plane stress perpendicular to the bending axis on the back-side of the strut in the Ni coating near the Ni/Al interface. Thus, the plot is comparable with the results from XRD shown in Figure 8. A decohesion in the cohesive zone instantaneously leads to a drop in the accommodated stress in the coating as found during the in situ bending tests at synchrotron (Figure 11a), especially for the untreated foam.

### 4.3. Fracture Energy

To evaluate the strength of the adhesion between the Ni and the Al phase, a detailed mechanical testing of the interface without effects from boundary conditions such as the strut geometry is necessary. Considering a smaller scale the necessary energy for stable crack growth along the interface is nearly two times higher for the pre-treated sample compared to the untreated one. The pre-treated beam fails more ductile and the crack follows just the big pore clusters. The connection between the coating and the base foam is intact, the material-fit and the form-fit connection outweighs the weakening by the pores in the Al phase. The pre-treatment process in our case has two effects onto the Al foam surface. One task is to clean it from oxides and to prepare the material for an improved material-fit connection. A second task is to roughen the surface to make a form-fit connection between the coating and the base foam surface possible. This roughening also seems to lead to the pore clusters. In contrast the first impression from the FIB tomography the material-fit and form-fit connection in the pre-treated specimen leads to better adhesion behaviour, although it introduces and still leaves large pore clusters in the Al phase.

The Al surface of an untreated foam is smooth on the length scale of a few μm and is of course covered by an oxide layer due to passivation. The Ni coating covers the smooth surface of the untreated Al foam nearly completely without significant porosity but maybe the oxide layer leads to worse bonding between Al and Ni.

However, the better bonding between Al and Ni due to the lack of oxide layers and the presence of a Zn layer as well as the Cu layer results in an improved material-fit connection. Additionally, the form-fit connection with its large shared surface between Al and Ni is an advantage over the pre-treated foam and triggers ductile failure with a higher energy absorption.

## 5. Conclusions

Our results clearly underline that a pre-treatment of the Al base foam before the electrodeposition of the Ni coating improves the strength of the connection at the interface, the shear stiffness of the interface and therefore the bending and buckling strength of the individual struts on the microscale of a foam, resulting in an increased strength and energy absorption capacity for the macroscopic foams.

Energy-dispersive XRD in the synchrotron allowed us to measure the stress depth distribution in the Ni coating of a Ni/Al hybrid foam and revealed residual compressive stresses before and a slightly reduced in-plane tensile stress perpendicular to the bending axis during in situ three-point bending near the Ni/Al interface. During the in situ test the moment of the interface decohesion was measured as a decrease in the stress accommodated in the Ni layer that is followed by a small plateau prior to fracture of the coating as seen in most specimens (Figure 8). This decrease in the accommodated stress was directly linked to the interface decohesion by FEM simulations and a postmortem analysis of the fracture process zone by SEM and optical microscopy.

A detailed analysis of the morphology of the interface by FIB implied a complex interplay between the mechanisms. The in situ micro bending tests revealed mechanisms of failure and energy dissipation prior to fracture.

Taking the three investigations into account, an assessment of the microstructure morphology can be made for the micro- and macroscopic behaviour under loading for the Ni/Al interface according to manufacturing processes. In both macroscopic and microscopic mechanical tests, the untreated foam sample was not as stable against decohesion of the Ni coating as the pre-treated foam sample. The pore clusters are the weakest points in the interface, but they withstand higher deformation energy during crack propagation than the smooth interface of the untreated sample. This can be traced back to two main effects:

- an improved material-fit
- a snap button-like form-fit connection

Knowing this, a further improvement of the Ni coating process is possible by an optimized pre-treatment. Maybe a shorter etching with NaOH or HNO_3_ might lead to lower roughness and consequently to less pore formation in the following coating process. To examine the effects of a shorter etching treatment on the interface structure a 2D FIB cross section becomes sufficient considering the aspects found during this investigation. Thus, several pre-treatments can be checked quickly and only few samples e.g., with the smallest pore sizes and yet large undercuts must be characterized by the more complex 3D FIB tomography and subsequent analyzed by microbeam bending. Hence, further optimization of the interface becomes more efficient and can still improve the energy absorption capacity of hybrid foams.

## Figures and Tables

**Table 1:** Shape parameters of the untreated (ut) and the pre-treated (pt) volume, with surface $S_{i}$ of the phase *i*, volume fraction of the pores $\phi_{\mathrm{pores}}$ and the shared surfaces $S_{i/j}$ of both phases i/j.

Specimen	ut	pt
$S_{Al}$/μm^2^	128.60	579.99
$S_{Ni}$/μm^2^	133.71	467.40
$S_{\mathrm{pores}}$/μm^2^	11.35	216.48
$\phi_{\mathrm{pores}}$/%	0.03	2.75
$S_{Al/Ni}$/μm^2^	125.48	415.45
$S_{Al/\mathrm{pores}}$/μm^2^	3.12	164.53
$S_{Ni/\mathrm{pores}}$/μm^2^	8.23	51.95

**Table 2:** Summary of the analyzed specimens of the untreated (ut) and pre-treated (pt) foam, the maximum force $F_{max}$ and the maximum mean in-plane bending stress $\sigma_{xx,max}$.

Specimen	ut1	ut2	ut3	pt1	pt2	pt3	ut	pt
pre-treatment	-	-	-	+	+	+	-	+
experiment	synchrotron EDDI						microbending	
$F_{max}$/N	17.6	15.0	11.3	53.1	25.2	26.4	-	
$\sigma_{xx,max}$/MPa	616.0	483.1	648.6	753.0	689.9	672.0	-	

**Table 3:** Dimensions an test results for the bending beams of the untreated (ut) and pre-treated (pt) specimen: beam thickness *t*, beam width *w*, cross-sectional area *A*, maximum force $F_{\max}$, maximum force normalized $F_{\max,\mathrm{norm}}$, critical energy $E_{\mathrm{crit}}$, critical crack length $a_{\mathrm{crit}}$.

Specimen	ut	pt
*t*/μm	9.57	7.29
*w*/μm	7.60	7.19
*A*/μm^2^	55.40	68.88
$F_{\max}$/mN	1.81	2.39
$F_{\max,\mathrm{norm}}$/mN μm^2^	0.0327	0.0348
$E_{\mathrm{crit}}$/mN μm	0.0954	0.1807
$a_{\mathrm{crit}}$/μm	0.2	1.5

## Data Availability

Data are contained within the article.

## Abbreviations

The following abbreviations and symbols are used in this manuscript:

EDDI	energy-dispersive diffraction
FEM	finite element method
FIB	focused ion beam
MMM	macro-meso-micro
SEM	scanning electron microscope
XRD	X-ray diffraction
AD	angular-dispersive
Al	aluminium
Cu	copper
ED	energy-dispersive
Ga	gallium
Ni	nickel
PU	polyurethane
Pt	platinum
pt	pre-treated
ut	untreated
Zn	zinc
*A*	cross-sectional area
$a_{crit}$	critical crack length
$d_{hkl}$	lattice spacing of plane hkl
*E*	elastic modulus
$E_{crit}$	critical energy
$F_{max}$	maximum force
$F_{max,norm}$	maximum force normalized
*J*	J-integral
*K*	stress intensity factor
ϕ	goniometer azimuth angle
ψ	goniometer polar angle
$\psi_{pores}$	volume fraction of the pores
$S_{Al}$	surface of Al
$S_{Ni}$	surface of Ni
$S_{pores}$	surface of pores
$S_{Al/Ni}$	shared surfaces of Al and Ni
$S_{Al/pores}$	shared surfaces of Al and pores
$S_{Ni/pores}$	shared surfaces of Ni and pores
$\sigma_{xx}$	in-plane bending stress
$\sigma_{xy}$	in-plane bending shear stress
$\sigma_{max,mean}$	maximum mean in-plane bending stress
*t*	beam thickness
τ	mean information depth
θ	incident beam angle (Bragg-Brentano goniometer)
*w*	beam width
ω	detector angle (Bragg-Brentano goniometer)
